# VARP and Rab9 Are Dispensable for the Rab32/BLOC-3 Dependent *Salmonella* Killing

**DOI:** 10.3389/fcimb.2020.581024

**Published:** 2020-12-16

**Authors:** Arda Balci, Virtu Solano-Collado, Massimiliano Baldassarre, Stefania Spanò

**Affiliations:** Institute of Medical Sciences, University of Aberdeen, Aberdeen, United Kingdom

**Keywords:** *Salmonella*, Rab32, VPS9-ankyrin-repeat-protein, Rab9, macrophages, BLOC-3

## Abstract

*Salmonella enterica* serovar Typhi (*S*. Typhi) is the causative agent of typhoid fever, a disease that kills an estimated 200,000 people annually. Previously, we discovered an antimicrobial pathway dependent on Rab32 and BLOC-3 (BRAM) that is critical to kill *S*. Typhi in murine macrophages. The BLOC-3 complex is comprised of the two sub-units HPS1 and HPS4 and exhibits guanine-nucleotide exchange factor (GEF) activity to Rab32. In melanocytes, Rab9 has been shown to interact with HPS4 and RUTBC1, a Rab32 GTPase activating (GAP) protein, and regulate the Rab32-mediated melanosome biogenesis. Intriguingly, Rab9-deficient melanocytes exhibit hypopigmentation, a similar phenotype to Rab32 or BLOC-3 deficient melanocytes. Additionally, VPS9-ankyrin-repeat-protein (VARP) has been shown to regulate melanocytic enzyme trafficking into the melanosomes through interaction with Rab32. Although Rab32, Rab9 and VARP are a part of melanogenesis in melanocytes, whether Rab9 and VARP are required for the BRAM mediated killing in macrophages is currently unknown. Here we showed that HPS4 is recruited to the *Salmonella-*containing vacuoles (SCV) and over-expression of BLOC-3 significantly increased Rab32-positive bacteria vacuoles. We found that SCV acquire Rab9, however over-expressing Rab9 did not change HPS4 localization on bacteria vacuoles. Importantly, we used shRNA to knock-down Rab9 and VARP in macrophages and showed that these proteins are dispensable for Rab32 recruitment to the SCV. Furthermore, we assessed the survival of *S*. Typhimurium in macrophages deficient for Rab9 or VARP and demonstrated that these proteins are not essential for BRAM pathway-dependent killing.

## Introduction

The Rab family represents the biggest member of Ras superfamily of small guanosine triphosphatases (GTPases) (Zhen and Stenmark, 2015). Small Rab GTPases are highly conserved proteins containing more than 60 members in mammals (Stenmark, 2009). Rab GTPases localize to a distinct organelle membranes or subcellular compartment and play a crucial role in orchestrating intracellular vesicle trafficking through tethering, docking, fusion and membrane exchange (Hutagalung and Novick, 2011). Like other small GTPases, Rab proteins shuttle between their GTP-bound active state to GDP-bound inactive state. The cycle is regulated by guanine-nucleotide exchange factors (GEF) that facilitate GTP loading and GTPase activating proteins (GAPs) that increase the intrinsic low GTP hydrolysis (Pylypenko et al., 2017). The small GTPase Rab32 with its GEF BLOC-3, play a vital role in the biogenesis of lysosomal related organelles (LROs) such as melanosomes (Bultema et al., 2012) and platelet dense granules (Ambrosio et al., 2012).

We have shown that the growth of the human-restricted pathogen *Salmonella enterica* serovar Typhi (*S*. Typhi) is restricted in mouse macrophages partially due to Rab32 and BLOC-3 (Spanò and Galán, 2012). In mouse macrophages, *S.* Typhi-containing vacuoles (SCV) have been shown to acquire Rab32 and this results in killing of the pathogen (Spanò, 2016). Interestingly, a broad-host pathogen *Salmonella enterica* serovar Typhimurium (*S*. Typhimurium) has evolved two effector proteins, GtgE, and SopD2, to counteract the Rab32/BLOC-3 dependent antimicrobial pathway (BRAM pathway). GtgE is a cysteine protease, which specifically targets Rab32, and SopD2 inactivates Rab32 through its GAP activity (Spanò and Galán, 2012; Spanò et al., 2016). In line with this, Rab32 or BLOC-3 deficient mice supported the replication of *S*. Typhi or a *S*. Typhimurium strain lacking GtgE and SopD2 (here after *S*. Typhimurium Δ*gtgE*Δ*sopD2*) (Spanò et al., 2016). Due to the role of Rab32 and BLOC-3 in the biogenesis of LROs, it has been hypothesised that Rab32 and BLOC-3 mediated bacterial restriction depends on trafficking of antimicrobial molecule(s) into the SCV, a mechanism that is inhibited by the broad-host *S*. Typhimurium.

Despite the importance of this host defence mechanism, how the BRAM pathway is regulated in macrophages and on the bacteria-containing vacuole in particular is largely unknown. Previous studies in melanocytes would suggest that Rab32 activity may be regulated by Rab9 and VPS9-ankyrin-repeat-protein (VARP), acting upstream and downstream of Rab32, respectively. In particular, in melanosomes, the BLOC-3 subunit HPS4 interacts with GTP-bound Rab9 and it has been suggested that this interaction is important for proper BLOC-3 complex localization. Interestingly, Rab9 also interacts with RUTBC1, the only known GAP for Rab32 (Kloer et al., 2010; Nottingham et al., 2011; Marubashi et al., 2015; Ohbayashi et al., 2017). The role of Rab9 in melanosome biogenesis is confirmed in Rab9 deficient melanocytes, which exhibited hypopigmentation due to mislocalization of melanosomal proteins (Mahanty et al., 2015). Similarly, an altered trafficking of melanosome cargoes has been observed in VARP knock-down melanocytes (Tamura et al., 2009). VARP is a Rab21-GEF protein and is known to interact with the GTP-bound form of Rab32 (Zhang et al., 2006; Tamura et al., 2009). Therefore, on melanosomes, Rab32 is activated in response to a Rab9 dependent recruitment of BLOC-3 and, in turn, regulates VARP. However, it is currently unknown if a similar regulation is in action on the bacterial containing vacuole and this needs to be tested.

In this study we confirmed that BLOC-3 plays a fundamental role in activating and localizing Rab32 on the SCV. However, using a combination of over-expression and RNA silencing experiments we demonstrate that neither Rab9 nor VARP are important for the BRAM-dependent *Salmonella* killing in macrophages.

## Materials and Methods

### Bacterial Strains and Primers

The wild-type *Salmonella* Typhimurium strain (SL1344) used in this study and *S.* Typhimurium SL1344 deletion strains (SB2527; *S*. Typhimurium Δ*gtgE*Δ*sopD2*) were described previously (Hoiseth and Stocker, 1981) and constructed *via* recombinant DNA and allelic exchange procedures as shown previously (Kaniga et al., 1994). Both *S.* Typhimurium SL1344 and SB2527 strains were engineered to chromosomally express codon-optimized *mCherry* gene by P22 transduction (a gift from Leigh Knodler; (Knodler et al., 2014)). All primers used in this study are as follows; sequences 5’ to 3’ f-VARP TCCTGCCAGTTCGAGTCCTAT, r-VARP AATGACGACAGCCTTTCCATC, f-Rab9a ATGGCAGGAAAATCGTCTCTTT, r-Rab9a GCATGGTAACAAAATGTCCGTCC, f-Rab9b ATGAGTGGGAAATCCCTTCTCT, r-Rab9b CCTGGGAGTCGAACTTGTTGG, f-Rab32 CGTGGGTAAGACGAGCATCAT, r-Rab32 CCCAGTTGAGAACTTTGAGGG, f-Rab38 CATGGCTTCGTAGGATGGTT, r-Rab38 AGATGGGGCTTCACAATGTC.

### Cell Culture

All cells used in this study were incubated at 37°C in a humidified 5% CO2 atmosphere. Immortalized-bone marrow derived macrophages (iBMDM) (BEI Recourses) and HeLa cells were maintained in Glutamax Dulbecco’s Modified Eagle’s Medium (DMEM) (Gibco) with 10% Fetal Bovine Serum (FBS) (Gibco). Cells were split after a wash with Dulbecco’s Phosphate Buffered Saline (Gibco) followed by trypsinization (Gibco). Cells were further seeded at the desired density. Alternatively, BMDM obtained from mice were isolated and differentiated as shown previously (Weischenfeldt and Porse, 2008). Briefly, mice were sacrificed by cervical dislocation and femur bones were removed. Muscles surrounding the bones were cleaned and bones were transferred into 70% ethanol for 1 min followed by a wash with PBS. Bones were cut from the two ends using sterile scissors and bone marrow cells were flushed out using 27-gauge needle (BD). Extracted cells were collected after centrifugation at 2,000 rpm for 5 min and cells were resuspended and maintained in DMEM high glucose 2 mM glutamax (Gibco), 10% FBS (Gibco), 20% L929 cell supernatant. Differentiated BMDM were used between day 6 and 10 after extraction.

### Transfection of HeLa Cells

HeLa cells were used at 70% confluency for transient transfection experiments. Briefly, cells were seeded at a density of 1x10^5^ on glass coverslips (#1 ThermoFisher Scientific) and left for incubation over-night. The DNA vectors were prepared in FBS free DMEM with polyethylenimine (PEI) at a ratio of 1:3 (DNA to PEI). The transfection mixture was then incubated 20 min at room-temperature and added to the cells. Transfected cells were used for the appropriate experiment 24 h after transfection. Following plasmids were used in the study; pCIneo-HA-HPS1 and pCIneo-Myc-HPS4 (gift of Juan S. Bonifacino), pEGFP, pEGFP-Rab9, and pCDNA3 (Stefania Spanò laboratory).

### Lentiviral Generation and shRNA Delivery

Lentivirus particles were produced by co-transfecting HEK293T cells at 70% confluency with 9 µg of pLKO shRNA with 9 µg pMLV-Gag-Pol and 0.9 µg pVSV-G in a 10 cm tissue-culture dish. The transfection was carried out using 3:1 ratio of PEI. The growth medium was replaced with DMEM containing 30% FBS the following day and the viral particles were collected 52 h post-transfection. Gene knock-down was performed as described previously (Taxman et al., 2010). Briefly, MISSION^®^ shRNA (Sigma-Aldrich) (Rab32-TRCN0000288450, TRCN0000102689, Rab9-TRCN0000100908, VARP-TRCN0000248745) were purchased as bacterial glycerol stocks and stored at -80°C according to the instructions from the manufacturer. Lentiviral particles expressing shRNAs were produced as described above and iBMDM cells were infected with the virus dilution 1:4. After 24 h, the medium was replaced with puromycin containing medium (5 µg/ml) to select the transduced cells. Selected cells were then used for the experiments. Alternatively, BMDM cells were infected with lentivirus at a ratio of 1:4 on either day 4 or 6 post-extraction. The cells were used for the experiments on day 7 or 9. Quantitative PCR was carried out in parallel to the experiments to validate the gene expression levels of the targeted gene.

### cDNA Synthesis and Quantitative PCR

cDNA synthesis was carried out using iScript™ cDNA synthesis kit (Bio-Rad). Briefly, 4 µl of 5x iScript reaction mix was mixed with 1µg of RNA and iScript reverse transcriptase. The final volume was brought up to 20 µl using nuclease-free water. The thermal cycler reaction was used as follow, priming 5 min at 25°C, reverse transcription 20 min at 46°C and inactivation of the enzyme 1 min at 95°C. The qPCR reaction was carried out using 96-well plate (Applied Biosystems) with a reaction mixture containing 2 µl of cDNA, 10 µl of SYBR green mix (Takara), 0.1 µl of 100 µM forward and reverse primer and 7.8 µl of nuclease free water. The thermal cycling was performed using StepOnePlus™ (Applied Biosystems) with following protocol; 95°C for 10 min, 40 cycles of 95°C for 15 s and 60°C for 1 min. Each targeted was quantified in triplicates and GAPDH was used as housekeeping gene. Differential gene expression was quantified using ΔΔCt method (Livak and Schmittgen, 2001). Primers for qPCR were selected from PrimerBank.

### Infection and Colony-Forming Unit Assay (CFU)

Over-night cultures of *Salmonella* strains were diluted 1:20 in 0.3 M NaCl LB-broth to induce the expression of the *Salmonella* pathogenicity island-1 (SPI-1) type-III secretion system. Bacterial cells were grown at 37°C with agitation in glass tubes until they reached OD_600_ 0.9 which corresponds to 10^9^cells/ml.

BMDM or iBMDM cells were seeded at a density of 1x10^5^/well in 24-well plates and infected with different *Salmonella* strains in HBSS at a MOI of 2. One-hour after infection, cells were washed three times with HBSS and incubated in appropriate growth medium containing 100 μg/ml gentamicin (Sigma) for 30 min to kill the extracellular bacteria. Cells were then washed and growth medium containing 5 μg/ml gentamicin was added to prevent reinfection (Patel et al., 2009). Cells were lysed at 1.5, 5-, and 24-h post-infection using 0.1% sodium deoxycholate (Sigma) in PBS to release the intracellular bacteria. Serial dilutions of lysates were plated on LB-agar plates supplemented with streptomycin (100 μg/ml) to quantify the *Salmonella* colonies formed on the plates.

### Fluorescent Microscopy

Cells were seeded at a density of 1x10^5^ onto glass coverslips (#1 ThermoFisher Scientific) and infected with *Salmonella* strains for 2.5 h. Then, cells were washed with PBS and fixed with 4% paraformaldehyde (PFA) (Agar Scientific) for 10 min at room-temperature. The cells were then permeabilized using permeabilization buffer (50 mM NH_4_Cl (Sigma), 0.2% Bovine serum albumin (BSA) (Sigma), 0.2% Triton X-100 (Sigma)) for 20 min at room-temperature. Antibodies used for immunofluorescence were diluted appropriately in permeabilization buffer and used as follows; monoclonal anti-Rab32 (Santa Cruz sc-390178) 1:200, polyclonal anti-Rab32 (Genetex GTX130477) 1:100, monoclonal anti-HPS4 (Santa Cruz sc-271425) 1:50 and DAPI (Thermofisher) 1:10,000. All samples were incubated with primary antibodies for 1 h followed by appropriate secondary antibody incubation for another hour at room-temperature. Coverslips were imaged using a Zeiss Axio Imager M1 with 60x oil objective. Acquired images were analyzed with ZEN software.

## Results

### BLOC-3 Sub-Unit HPS4 Is Recruited to the SCV in HeLa Cells

Rab proteins need to be loaded with GTP to associate with membranes and we have previously shown that Rab32 recruitment to the SCV depends on its GEF, BLOC-3 (Spanò and Galán, 2012). However, the sub-cellular localization of BLOC-3 and consequently the compartment where Rab32 is activated in infected cells is still unknown.

**Figure 1:**
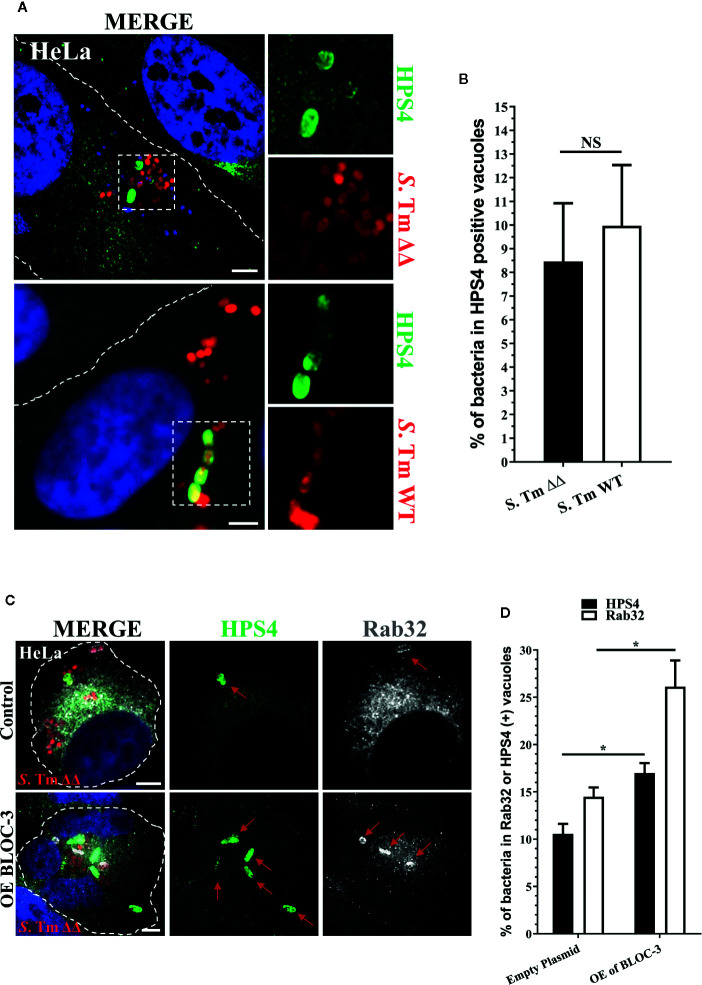
BLOC-3 sub-unit HPS4 is recruited to the *Salmonella*-containing vacuoles (SCV). **(A)** HeLa cells were infected with *mCherry S*. Typhimurium Δ*gtgE*Δ*sopD2* (ΔΔ) or WT for 2.5 h, fixed and stained with monoclonal anti-HPS4 antibody (green). **(B)** The percentage of *S*. Typhimurium in HPS4-positive vacuoles were analyzed by immunofluorescence and the ± standard deviation of three independent experiments are shown. At least 100 bacteria were counted in each experiment. **(C)** HeLa cells either over-expressing BLOC-3 complex or empty plasmid (control) were infected with *S*. Typhimurium Δ*gtgE*Δ*sopD2*, fixed at 2.5-h post-infection and co-stained with anti-HPS4 (green) and anti-Rab32 (gray) antibodies. Images were analyzed using fluorescence microscopy. **(D)** The percentage of bacterium in HPS4 or Rab32 positive vacuoles were analyzed by immunofluorescence and the ± standard deviation of three independent experiments are shown. (Student’s t test; *p < 0.05, NS, Non-significant), *S*. Tm, *Salmonella* Typhimurium; Scale bar: 5μm.

Therefore, we investigated the localization of BLOC-3 in HeLa cells infected with *S*. Typhimurium. At 2.5 h post infection, we were able to detect the BLOC-3 subunit HPS4 on 7%–8% of SCV (**Figures 1A, B**). Notably, the percentage of HPS4 positive SCVs did not change when HeLa cells were infected with *S*. Typhimurium Δ*gtgE*Δ*sopD2*, suggesting that the localization of HPS4 is an event that happens upstream and independent of the localization of Rab32. The low percentage of HPS4 positive vacuoles indicates that BLOC-3 recruitment to the vacuole is probably transient. Indeed, HPS4 recruitment increased when the two BLOC-3 subunits HPS1 and HPS4 were co-overexpressed in HeLa cells (**Figures 1C, D**). More importantly, BLOC-3 overexpression also increased the percentage of Rab32 positive vacuoles, confirming its key role in Rab32 localization to SCV and in activation of the BRAM pathway (**Figures 1C, D**).

Overall, these data suggest that BLOC-3 does localize, at least transiently, on the SCV and that over-expression of BLOC-3 results in increased amount of Rab32 on the SCV.

### Rab9 Function Is Redundant for Rab32 Recruitment to the SCV and BRAM Pathway

**Figure 2:**
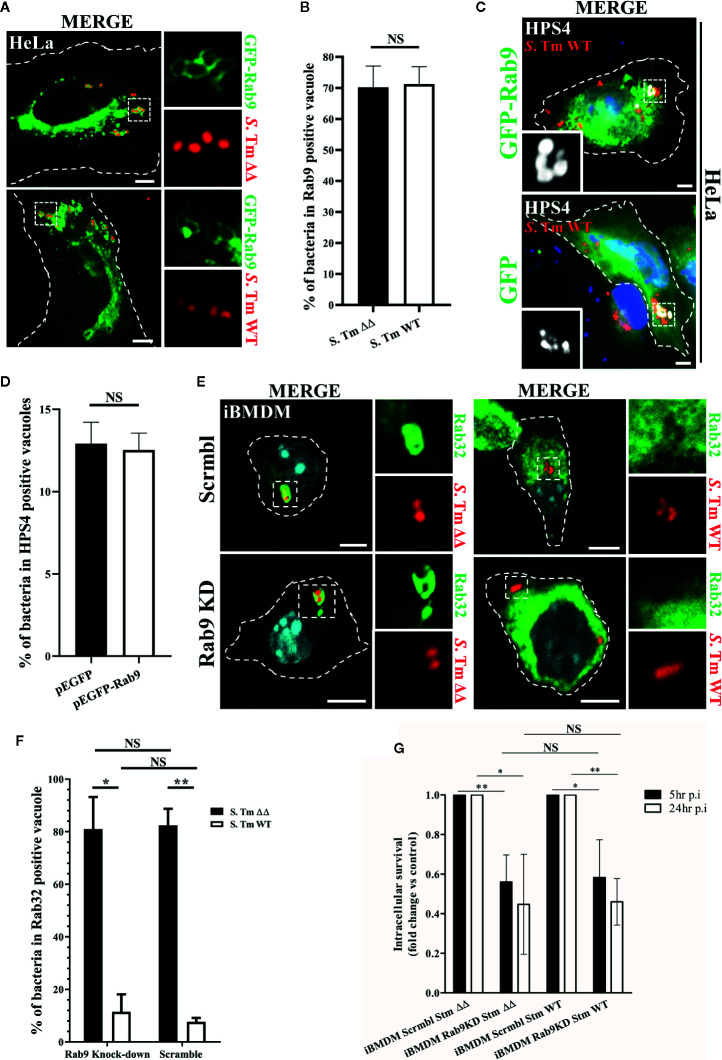
Rab9 does not regulate Rab32 recruitment to the *Salmonella*-containing vacuoles (SCV) and its function is not required for Rab32/BLOC-3 dependent killing. **(A)** GFP-Rab9 transfected HeLa cells were infected with *mCherry S*. Typhimurium Δ*gtgE*Δ*sopD2* (ΔΔ) or WT for 2.5 h, fixed and images were acquired using fluorescent microscopy. **(B)** The percentage of *S*. Typhimurium in Rab9-positive vacuoles at 2.5 h post-infection is shown with ± standard-deviation of three independent experiments. **(C)** HeLa cells transfected with either GFP or GFP-Rab9 were infected with *mCherry S*. Typhimurium WT for 2.5 h. Cells were then fixed, stained with monoclonal anti-HPS4 (gray) antibody and analyzed by fluorescence microscopy. **(D)** The percentage of *S*. Typhimurium in HPS4-positive vacuoles at 2.5 h post-infection is shown with ± standard-deviation of three independent experiments. **(E)** iBMDM cells knocked down for Rab9 (Rab9 KD) and control (Scrmbl) were infected with mCherry *S*. Typhimurium Δ*gtgE*Δ*sopD2* or WT for 2.5 h, fixed and stained with anti-Rab32 (green). Images were acquired using fluorescent microscopy **(F)** The percentage of *S*. Typhimurium in Rab32-positive vacuoles is shown with the ± standard-deviation from three independent experiment. **(G)** iBMDM cells depleted for Rab9 (Rab9 KD) and control cells (Scrmbl) were infected with *S*. Typhimurium Δ*gtgE*Δ*sopD2* or WT, lysed at the indicated time points and colony-forming units were calculated. Values are presented as fold change compared to the initially internalized (1.5 hpi). Error bars indicate standard-deviation of five independent experiments. For the non-normalized values see **Figure S2**. (Student’s t test; *p < 0.05, **p < 0.01) *S*. Tm, *Salmonella* Typhimurium; KD, Knock-down; CFUs, Colony-forming units; Scale bar: 5μm; NS, non statistically different.

Earlier studies showed that the *S*. Typhimurium effector protein SifA recruits and sequesters Rab9 on the SCV, a mechanism that prevents the trafficking of lysosomal enzymes to the pathogen-containing vacuole (McGourty et al., 2012). Interestingly, Rab9 has been also shown to interact with HPS4 protein and it is thought to regulate BLOC-3 localization on melanosomes (Kloer et al., 2010; Ohbayashi et al., 2017). Thus, we hypothesized that Rab9 may act in an analogous manner on the SCV by mediating the recruitment of BLOC-3. To test this possibility, we transfected HeLa cells with GFP-Rab9, infected them with *S*. Typhimurium::*mCherry* and looked at Rab9 and HPS4 localization. In agreement with McGourty et al., we confirmed that GFP-Rab9 is recruited to the SCV (**Figure 2A**). Interestingly, the percentage of Rab9 positive vacuoles was not dependent on Rab32 activation as significant differences were not detected in cells infected with *S*. Typhimurium WT compared to *S*. Typhimurium *ΔgtgEΔsopD2* (**Figure 2B**). We also looked for HPS4 localization in *S*. Typhimurium WT infected HeLa cells expressing either GFP or GFP-Rab9 (**Figure 2C**) and showed that over-expression of GFP-Rab9 did not increase the percentage of vacuoles positive for HPS4 (**Figure 2D**), suggesting that HPS4 recruitment on the SCV is independent of Rab9. In view of these results, we investigated whether depletion of Rab9 in macrophages affects the BRAM-pathway-dependent *Salmonella* killing.

Firstly, we analyzed whether depletion of Rab9 from iBMDMs affected Rab32 trafficking to the SCV. Briefly, iBMDMs were transduced with lentivirus encoding an shRNA targeting Rab9, cells were then selected with puromycin and knockdown efficiency was assessed by qPCR (~%85) (**Figure S1a**). To confirm that Rab9a is the main Rab9 isoform in macrophages and to exclude a Rab9b compensatory function after Rab9a knockdown, we also determined the levels of Rab9b. Our qPCR analysis showed that Rab9b is barely detected in macrophages (**Figure S1b**) and its expression does not increase significantly in macrophages upon Rab9a depletion (**Figure S1c**). Rab9 deficient macrophages were then infected with *mCherry S*. Typhimurium WT or *S.* Typhimurium Δ*gtgE*Δ*sopD2* for 2.5 h, fixed and stained with anti-Rab32 antibody (green). Cells were then imaged using fluorescent microscopy. Our results demonstrated that Rab32 trafficking to the SCV is not affected in macrophages deficient for Rab9 (**Figure 2E**). Moreover, the percentage of *S*. Typhimurium in Rab32 positive vacuoles is not significantly different in Rab9 knock-down cells compared to control cells (**Figure 2F**). These results suggest that Rab9 does not regulate BLOC-3 dependent Rab32 localization to the SCV in macrophages.

Secondly, we used a colony-forming unit assay to assess whether *S*. Typhimurium survival is affected in macrophages with decreased Rab9 expression. As showed in **Figure 2G**, Rab9 depletion marginally affects *S.* Typhimurium intracellular survival, probably because interferes with the Salmonella induced filaments (SIF) formation acting on the sifA-SKIP axis (Jackson et al., 2008; McGourty et al., 2012). However, the effect on intracellular survival is equally present both in absence (*S.* Typhimurium WT) or in presence (*S.* Typhimurium *ΔgtgEΔsopD2*) of the BRAM pathway, indicating that Rab9 effect is independent of Rab32 antimicrobial activity.

### VARP Is Dispensable for Rab32 Trafficking to the SCV and *S*. Typhimurium Killing in Macrophages

Our data clearly demonstrate that Rab32 upstream regulation is different in melanosomes and the SCV. We then asked if this difference was also present in the downstream regulation, by analysing the role that the Rab32 effector, VARP, plays in SCV function.

**Figure 3:**
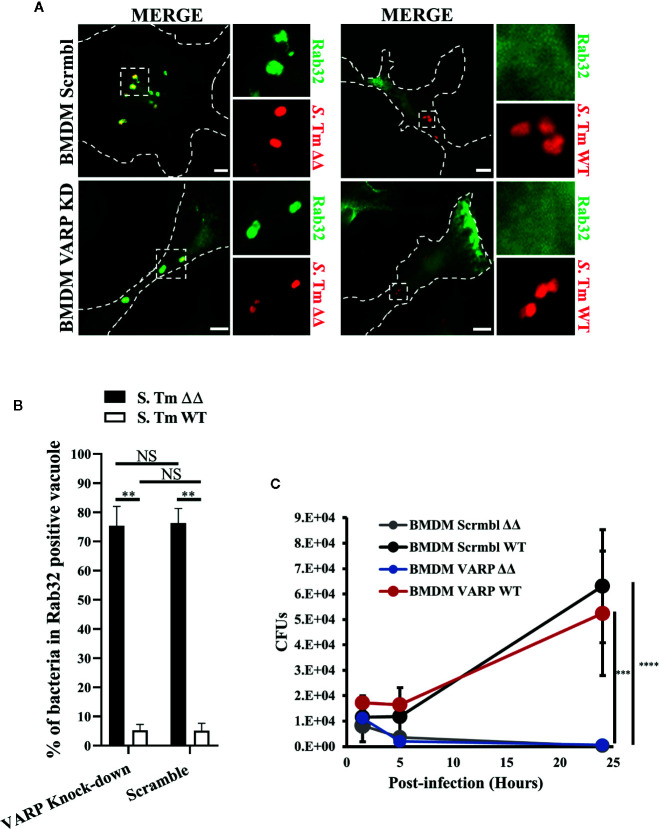
VPS9-ankyrin-repeat-protein (VARP) is not required for Rab32 trafficking to *Salmonella*-containing vacuoles (SCV) and its role in *Salmonella* killing is dispensable. **(A)** BMDM cells transduced with control (Scramble) or VARP shRNA (VARP KD) were infected with *mCherry S*. Typhimurium Δ*gtgE*Δ*sopD2* (ΔΔ) or WT for 2.5 h, fixed and stained with anti-Rab32 (green). Images were acquired using fluorescent microscopy. **(B)** The percentage of *S*. Typhimurium in Rab32-positive vacuoles were quantified in scramble and VARP KD cells and the ± standard-deviation of three independent experiments are shown. **(C)** BMDM cells depleted for VARP were infected with *S*. Typhimurium ΔΔ or WT, lysed at the indicated time points and colony-forming units were calculated. Values are standard-deviation of CFUs at each time points from three independent experiments. *S*. Tm, *Salmonella* Typhimurium; KD, Knock-down; CFUs, Colony-forming units; Scale bar: 5μm. (Student’s test; **p < 0.01, ***p < 0.001, ****p < 0.0001, NS, non statistically different).

VARP is a Rab32 and Rab38 interacting protein that regulates the trafficking of melanogenic enzymes to melanosomes (Tamura et al., 2009). We have demonstrated that Rab32 but not Rab38 removal is sufficient to observe an inhibitory effect in macrophage killing, indicating that Rab32 is the main Rab GTPase restricting *Salmonella* growth in bone marrow derived macrophages (BMDM) (Spanò and Galán, 2012). In fact, Rab38 is present in very low abundance in BMDM, (**Figure S1D**) and its expression does not increase after Rab32 knockdown (**Figure S1E**). Therefore, we hypothesized that Rab32 and VARP interaction might be crucial for the delivery of the antimicrobial cargo into the SCV. To test if Rab32 trafficking is impaired in the absence of VARP, we knocked-down VARP in BMDM using shRNA treatment and confirmed that VARP expression is significantly reduced in these cells (**Figure S1F**). We showed that VARP is not involved in Rab32 trafficking to the SCV. In fact, both VARP depleted and control cells were able to recruit Rab32 to the SCV (**Figure 3A**). Moreover, we measured the percentage of bacteria in Rab32 positive vacuoles and showed that there was no significant difference between VARP knock-down and control BMDMs (**Figure 3B**).

Next, we investigated if depleting VARP from macrophages impairs the BRAM-dependent *Salmonella* killing. To test this, we infected both control (Scrmbl) and VARP knock-down (VARP KD) BMDM with *S.* Typhimurium Δ*gtgE*Δ*sopD2* and *S.* Typhimurium WT, lysed the cells at indicated times (1.5, 5, and 24 h) and enumerated colony-forming units. Our results revealed that, as expected, *S.* Typhimurium WT, which can neutralize the BRAM pathway, survived in both scrmbl and VARP KD cells at 24 h post-infection (**Figure 3C**). On the contrary, both scrmbl and VARP KD macrophages were able to clear the *S.* Typhimurium Δ*gtgE*Δ*sopD2* infection with similar efficiency (**Figure 3C**). These results indicate that VARP does not regulate the BRAM pathway and *S.* Typhimurium survival in macrophages is independent of VARP protein.

Altogether, our results indicate that Rab32 localization and function on the SCV is regulated differently in melanosome and the SCV and that there are unknown important players regulating the BRAM pathway and therefore, bacterial clearance in macrophages.

## Discussion

Studies from our laboratory and others, have shown that in macrophages, the BRAM pathway controls not only infection by bacterial pathogens such as *Salmonella* Typhi, *Staphylococcus aureus*, *Listeria monocytogenes*, and *Burkholderia pseudomallei* (Seto et al., 2011; Spanò and Galán, 2012; Li et al., 2016; Hu et al., 2019) but it also has an important role in controlling *Candida albicans* infection in mice (Baldassarre et al., 2020). However, nothing is known about how the BRAM pathway is regulated and what other proteins are part of this important antimicrobial pathway. Here, we investigated the direct role of Rab9 and VARP in regulation of the Rab32 and BLOC-3-dependent antimicrobial pathway in macrophages.

The small GTPase Rab9 mainly localizes on late endosomes and regulates the trafficking of mannose-phosphate receptors (MPR). However, a number of studies reported that Rab9 functions in melanosome biogenesis by localizing to the melanosomes and is thought to regulate Rab32 through recruitment of BLOC-3 and RUTBC1 (Lombardi et al., 1993; Kloer et al., 2010; Nottingham et al., 2011; Mahanty et al., 2015; Marubashi et al., 2015; Ohbayashi et al., 2017). Similarly, we hypothesized that Rab9 may function as a regulator of the BRAM pathway by controlling BLOC-3 localization and Rab32 activation on the SCV. To test our hypothesis, we first addressed the sub-cellular localization of Rab9 in *Salmonella* infected cells using fluorescence microscopy. We confirmed that GFP-Rab9 is recruited to the SCV. Indeed, previous findings revealed that Rab9 is sequestered by *S.* Typhimurium effector protein SifA, which binds to host SKIP protein to form a “sink” for Rab9 on the SCV membranes, thereby attenuating retrograde transport of MPRs and lysosomal function (Jackson et al., 2008; McGourty et al., 2012). We have also showed that HPS4 is indeed recruited to the SCV and its over-expression significantly increases Rab32 positive SCV. However, HPS4 localization on the SCV does not seem to depend on Rab9 (**Figures 2C, D**).

To better understand the molecular relationship of Rab9 and BLOC-3, we knocked-down Rab9 in mouse macrophages and revealed that Rab9 deficient cells were still able to clear *S*. Typhimurium Δ*gtgE*Δs*opD2* suggesting that, in mouse macrophages, Rab9 does not affect Rab32 antimicrobial activity (**Figure 2G** and **Figure S2**). To further support this conclusion, we also found that Rab32 was successfully recruited to SCV in both control and Rab9 knock-down cells. Collectively, these results indicate that Rab9 does not control Rab32 recruitment or Rab32-dependent clearance of *Salmonella* in macrophages.

A recent study has confirmed the important role of BLOC-3, Rab32/38 and Rab9 in melanogenesis. They showed that Rab9 regulates Rab32 localization on melanosomes, but demonstrated that the interaction between HPS4 and Rab9 is dispensable for the melanogenesis (Ohishi et al., 2019). We found that Rab32 levels are almost 50 times higher in macrophages compared to Rab38, and knock-down of Rab32 does not increase Rab38 mRNA levels, indicating that Rab32 is the main player in macrophages. We therefore assessed the role of the Rab32 interacting protein VARP, which has been previously described to be a GEF for Rab21 (Zhang et al., 2006; Tamura et al., 2009). Depletion of VARP in melanocytes has been shown to reduce melanocytic enzyme transport, thereby impairing melanosome biogenesis (Tamura et al., 2009). Furthermore, we found that depletion of VARP does not impair Rab32 recruitment to the SCV. In agreement with these data, we also demonstrated that macrophages deficient for VARP are able to clear *S*. Typhimurium Δ*gtgE*Δ*sopD2* infection confirming that VARP is not required for the Rab32-dependent killing of *Salmonella*.

All together our results indicate that the BRAM pathway is not controlled by neither Rab9 nor VARP suggesting that that Rab32 is controlled through a different regulation circuit during infection.

Rab32 belongs to a group of 20 Rabs that were present in the last eukaryotic common ancestor (LECA) (Klöpper et al., 2012), so it is expected to control some basic cell function. At the same time, it is clear that Rab32 and BLOC-3 proteins have cell-type specific functions. For example, Rab32 acts as a PKA anchoring protein in non-melanogenic cells and has been shown to localize mainly to mitochondria in fibroblasts, HeLa, and COS7 cell lines whereas no-colocalization of Rab32 with mitochondria was seen in melanogenic cells (Alto et al., 2002; Wasmeier et al., 2006; Hirota and Tanaka, 2009). Furthermore, Rab32 accumulation in neural cells upon acute brain inflammation has been demonstrated to signal as an unfolded-protein response (Haile et al., 2017). If these more “modern” and cell specific functions of Rab32 have evolved different controlling circuits in different cell systems is a remarkably interesting question.

It is therefore likely that molecular modulators of Rab32 and BLOC-3 are different in mouse macrophages compared to melanocytes. One tempting hypothesis is that macrophages contains a Rab32 effector non-present in other cell types. Interestingly, during the revision of this manuscript, the Galán group showed that Rab32 binds to IRG1 and regulates the delivery of itaconic acid to the SCV (Chen et al., 2020). However, even if the elusive antimicrobial agent delivered by BRAM pathway may have been identified, there are still a number of important questions that are waiting to be answered. For example, IRG1 interacts with Rab32 independently of its nucleotide state (Chen et al., 2020), but the importance of BLOC-3 and therefore Rab32 activation in the bacterial killing is undeniable. So why Rab32 needs to be activated on the SCV? Moreover, the question on what keeps the active Rab32 on the vacuole and the exact role of BLOC-3 in IRG1 recruitment is still open. Finally, it is important to point out that *S.* Typhi survives better in HPS4 knock-out macrophages compared to IRG1 knock-out cells (Chen et al., 2020), suggesting that IRG1 and itaconate are not the only effectors downstream of Rab32.

## Author’s Note

This paper is part of SS’s scientific legacy and this would have not been possible without her intelligence, vision and persistence. A dreadful destiny has snatched her from us too early, but her discoveries and ideas are living and flourishing.

## Data Availability Statement

The original contributions presented in the study are included in the article/**Supplementary Material**. Further inquiries can be directed to the corresponding author.

## Author Contributions

AB performed the experiments and wrote the manuscript. VS-C performed experiments and revised the final version of the manuscript. SS and MB were involved in the design of the study, interpretation, and supervision of the study. MB wrote and revised the manuscript. All authors contributed to the article and approved the submitted version.

## Funding

This work was supported by the Wellcome Trust (Seed Award 109680/Z/15/Z), the European Union’s Horizon 2020 ERC consolidator award (2016-726152-TYPHI), the BBSRC (BB/N017854/1), the Royal Society (RG150386), and Tenovus Scotland (G14/19) to SS.

## Conflict of Interest

The authors declare that the research was conducted in the absence of any commercial or financial relationships that could be construed as a potential conflict of interest.
