# Supplementary material for: VARP and Rab9 Are Dispensable for the Rab32/BLOC-3 Dependent Salmonella Killing
Source: Front Cell Infect Microbiol. 2020 Dec 16;10:581024. doi: 10.3389/fcimb.2020.581024 (PMC7772198; doi:10.3389/fcimb.2020.581024)
Supplement: Supplementary file 1 [file DataSheet_1.docx]

**
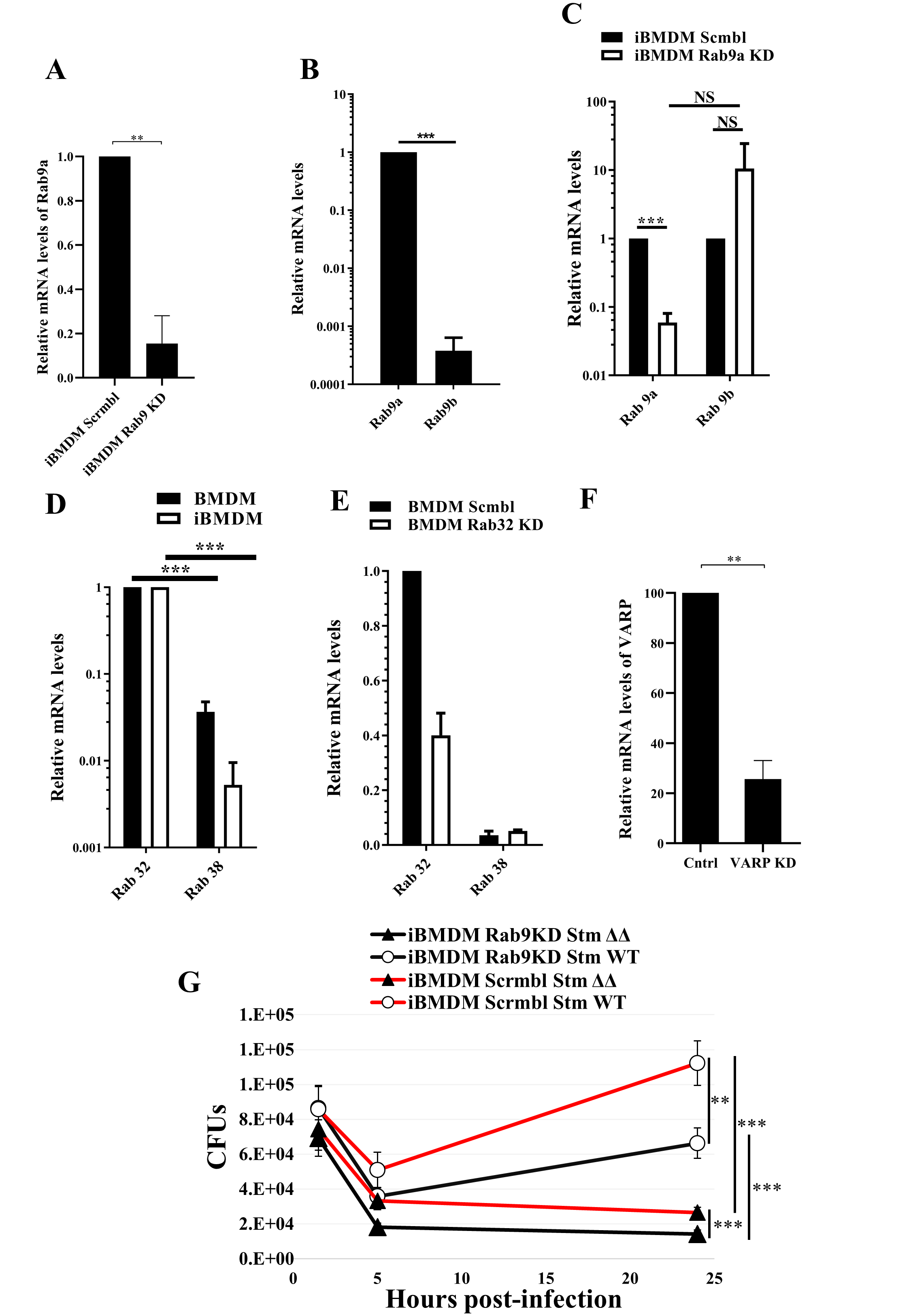
Figure S1. Relative mRNA levels of Rab9a, Rab9b, VARP, Rab32 and Rab38. (A)** qPCR quantitative expression analysis of iBMDM scrmbl and Rab9a knock-down cells. Relative mRNA levels of Rab9a is shown as ± standard-deviation of three independent experiments. **(B)** Relative mRNA expression levels of Rab9b in mouse macrophages is shown as ± standard-deviation of three independent experiments. **(C)** Relative mRNA levels of Rab9b in Rab9a KD macrophage cells is shown as ± standard-deviation of three independent experiments. **(D)** Relative mRNA levels of Rab38 in BMDM or iBMDM cells are shown as ± standard-deviation of three independent experiments. **(E)** Relative mRNA levels of Rab38 in Rab32 KD macrophage cells is shown as ± standard-deviation of two independent experiments. **(F)** qPCR quantitative expression analysis of BMDM scrmbl and VARP knock-down cells. Relative mRNA levels of VARP is shown as ± standard-deviation of three independent experiments. (Student’s t test; * p<0.05, ** p<0.01, *** p<0.001)**,** KD: Knock-down,


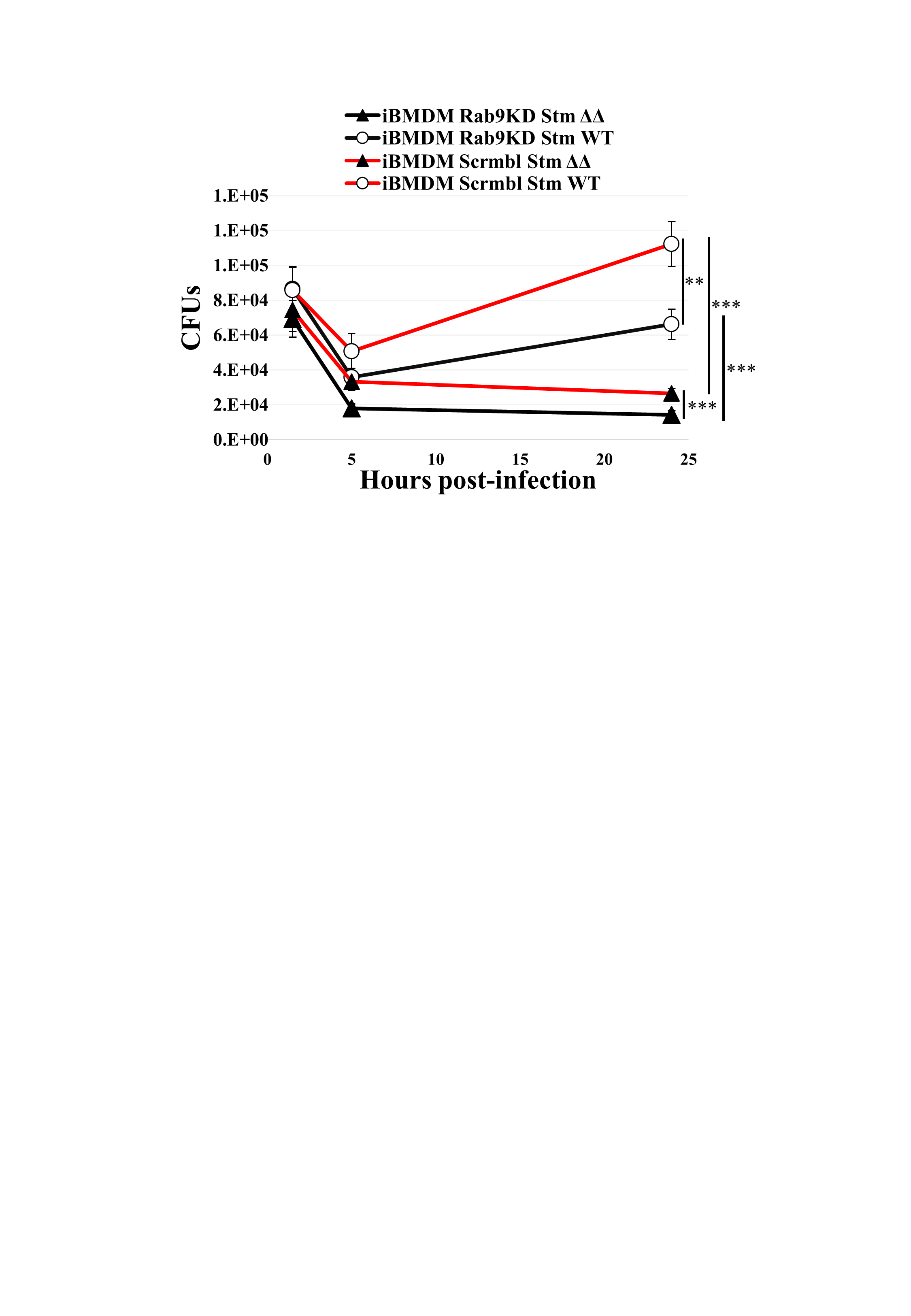
**Figure S2. Survival of Salmonella in Rab9KD macrophages**. iBMDM cells depleted for Rab9 were infected with *S*. Typhimurium ΔΔ or WT, lysed at the indicated time points and colony-forming units were calculated. Values are means ± SEM of five independent experiments performed in triplicates. (Student’s t test; * p<0.05, ** p<0.01, *** p<0.001) *S*. Tm: *Salmonella* Typhimurium**,** KD: Knock-down, CFUs: Colony-forming units.
